# Increase in the Risk of Respiratory Disorders in Adults and Children Related to Crop-Growing in Niger

**DOI:** 10.1155/2016/9848520

**Published:** 2016-12-21

**Authors:** Ali Mamane, Jean-François Tessier, Ghislaine Bouvier, Roger Salamon, Pierre Lebailly, Chantal Raherison, Isabelle Baldi

**Affiliations:** ^1^ISPED, Equipe Epidémiologie des Cancers et des Expositions Environnementales, Centre Inserm U1219, Bordeaux Population Health Center, Université de Bordeaux, Bordeaux, France; ^2^ISPED, Centre Inserm U1219, Bordeaux Population Health Center, Université de Bordeaux, Bordeaux, France; ^3^Service d'Information Médicale, Centre Hospitalier Universitaire de Bordeaux, Bordeaux, France; ^4^UMR 1086 Cancers et Préventions, Université de Caen Basse-Normandie, Caen, France; ^5^Service des Maladies Respiratoires, Centre Hospitalier Universitaire de Bordeaux, Bordeaux, France; ^6^Service de Médecine du Travail, Centre Hospitalier Universitaire de Bordeaux, Bordeaux, France

## Abstract

*Background and Objective.* Environmental factors are an increasing concern for respiratory health in developing countries. The objective of this study was to investigate whether Nigerien people living in cultivated areas have more respiratory symptoms than those living in pastoral areas.* Method.* A cross-sectional study was conducted in 2013 in two populations during the rainy season when land is cultivated. Environmental factors including pesticide use and respiratory symptoms were collected in adults and children during face-to-face interviews. Multivariate analysis between exposures and symptoms was performed in children and in adults separately.* Results.* The study included 471 adults and 229 children. Overall, none of the households reported the use of pesticides for agricultural purposes. However, 87.2% reported the use of insecticides at home. Multivariate analysis showed that people living in agricultural areas compared to those in pastoral areas had an increased risk of respiratory symptoms in adults (wheezing, dyspnea, sudden shortness of breath, and cough without fever) and in children (cough without fever). The use of insecticides showed no effect on respiratory symptoms after adjustment.* Conclusion.* This first epidemiological study on the environment and respiratory health conducted in Niger demonstrates a significant relationship between respiratory manifestations and the agricultural characteristics of the living area. However only the effect of insecticides in the home on respiratory health was observed.

## 1. Introduction

In recent decades, environmental factors such as occupational exposures have become a concern for respiratory health in the developing countries. Agriculture, the main industry in these countries, now has recourse to intensive use of pesticides in order to increase global food production. Pesticides could be herbicides, fungicides, acaricides, rodenticides, and molluscicides [1]. In the meantime, the growing use of these chemicals has raised questions about the risks for population health [2]. It is well known that people working or living on farms or in their vicinity can be exposed to serious environmental health risks [3–6]. The World Health Organization (WHO) considers that environmental factors are a root cause of an estimated one-quarter of the global burden of disease, rising to more than one-third in very poor regions such as Sub-Saharan Africa [7–9]. The populations of these countries are more vulnerable because of the lack of regulations, the absence of health-monitoring systems, and inadequate information on the precautions required with regard to environmental factors [3, 6, 9]. Among the 626 million people living in the Sub-Saharan region of Africa, 61% are directly involved in agriculture. Tropical insects and parasites are among the biggest challenges faced by agriculture and populations in this part of the world, leading to an increasing use of pesticides [10]. Although there is rising concern about the health impact of these substances, epidemiological data are very scarce in these countries [6, 11, 12].

It is well known that, in developing countries, farmers are supplied with unregistered chemicals or those banned from sale and that the low literacy rate prevents them from being aware of the health risks (WHO) [13]. A pragmatic inventory conducted in 2012 near the Benin, Nigeria, and Ghana border showed that numerous pesticide products were sold illegally on markets by people based in neighboring countries (unpublished personal data). Indeed, most of these products are banned by the Sahelian Pesticide Committee which comprises members from nine countries in the Sahel and therefore have no legal registration. Moreover, dangerous compounds were identified such as highly toxic organophosphates.

In Niger, respiratory diseases have been shown to be a major public health issue. With 34,000 deaths per year, lower respiratory diseases were ranked first by WHO among the causes of death in the population in 2002 and 2nd in the medical consultations after malaria [14]. Since the lungs are the first organs in contact with airborne pollutants and because of the high proportion of farmers in the Nigerien population, we decided to explore the hypothesis that pesticides could influence their respiratory health.

The aim of our study was to compare the prevalence of respiratory symptoms in two groups of people, one living in a subtropical area largely devoted to crop farming and potentially exposed to environmental factors such as pesticides and the other one living in a pastoral area with a priori lower exposure to these factors.

## 2. Materials and Methods

### 2.1. Sampling Design and Data Collection

The study was conducted in Niger, the largest country in West Africa, covering about 1,270,000 km^2^, of which 80 percent are desert. Farming is concentrated near the southern border with Nigeria (near the Niger River) and in the southeast near Lake Chad [15]. Niger's agriculture is mainly based on traditional subsistence crops (millet, sorghum, cassava, and rice) and livestock (camels, goats, sheep, and cattle) grown in very small farms. The majority of the 17 million inhabitants, whose life expectancy is 54 years, are in a chronic state of food insecurity [15, 16]. The economy of Niger relies largely on its agriculture which produces about 41% of the country's gross domestic product (GDP) (27% from crops, 10% from livestock, and 4% from fisheries and forests) [16]. Owing to the climatic conditions, Niger is one of the hottest and driest countries in the world but rainfall is more frequent in the South. Thus, in order to optimize crop yield during the rainy season (from May through September), pesticides are widely used in the agricultural areas of the South. Most of them are used to fight against grasshoppers, crickets, and locusts, and the inhabitants also use insecticides in their homes to fight against disease vectors.

**Figure 1:**
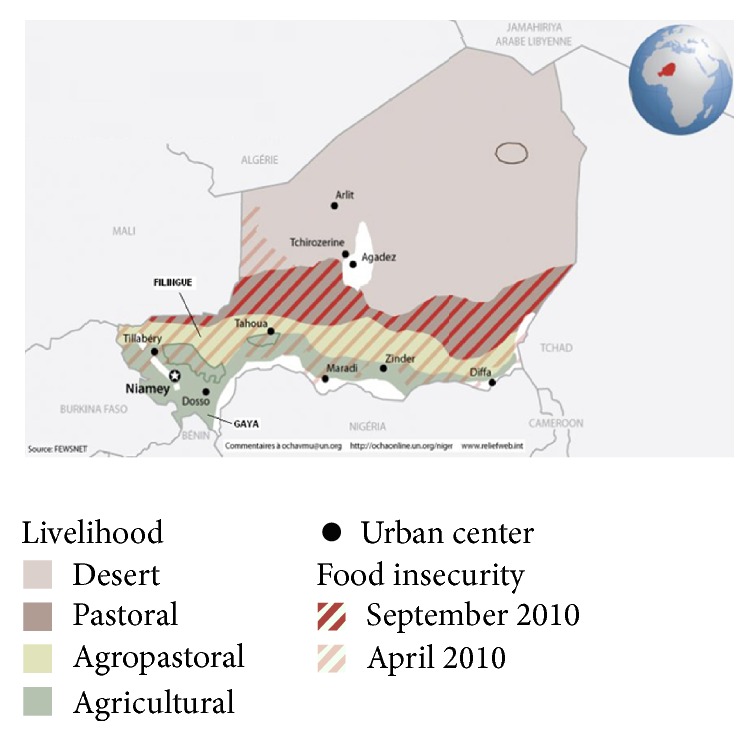
Agropastoral (Filingué) and agricultural (Gaya) areas selected for the study in Niger (source: http://ochaonline.un.org/niger).

We selected one desert area with pastoral activity and no use of pesticides on the rare crops grown (Filingué, Tillabéry Region) and one area where the subtropical climate favors crop-growing (Gaya, Dosso Region) (Figure 1). All the households living in Filingué and Gaya were invited to participate in the study.

The survey was a cross-sectional study which was performed in 2013 during the rainy season (August 26–September 25), when pesticides are usually sprayed on crops in Gaya.

The survey was based on the basic health units (*Centres de Santé Intégrés*) of Niger with the support of the responsible nurses. Two investigators who were well known to the inhabitants of the area were recruited and trained before the study. In agreement with the heads of the villages, door-to-door visits helped to identify and contact all households in both areas, that is, groups of people living together in the same dwelling and sometimes including several nuclear families.

A person in the household was eligible if he or she met the following inclusion criteria: residence in the zone, aged seven years or more, and present in the village during the investigation period. Written informed consent was requested from the heads of the household.

The questionnaire, which was adapted in part from the ISAAC respiratory study [17], was tested so that the wording of each question remained unmodified when translated into local languages (*Hausa* in Filingué and* Djerma* in Gaya).

The main questions taken from ISAAC were for adults: Have you ever had asthma? Have you ever had wheezing or whistling in the chest at any time in the past? Have you ever had a problem with sneezing, or a runny, or blocked nose when you DID NOT have a cold or the flu? For children: Has your child ever had asthma? Has your child ever had wheezing or whistling in the chest at any time in the past? Has your child ever had a problem with sneezing, or a runny, or blocked nose when he/she DID NOT have a cold or the flu?

Smoking was assessed according to three categories never smokers, current smokers, and former smokers (individuals who had stopped smoking at least one month prior to the examination). Due to their limited numbers, current and former smokers were pooled in the analysis.

Data collected during a face-to-face interview focused on the household environmental characteristics, on the individual characteristics of each household member, and on respiratory health data: asthma confirmed by a health professional, wheezing, out-of-breath sensation, cough, nocturnal awakening by a fit of coughing, cough without fever, and ocular or nasal irritation. Further questions were related to smoking habits.

Unfortunately in order to evaluate the potential respiratory effect of pesticides and insecticides exposure, it was not possible in the local context of this preliminary explorative study to perform environmental pesticides measures which could reflect populations chronic exposure. It is the reason why we limited our evaluation of pesticides and insecticides contact by a report of their use by the studied populations.

### 2.2. Data Analysis

Data were analyzed with the SAS 9.3 software. Descriptive statistics were carried out to describe the background characteristics of the study sample.

Respiratory symptoms were described and compared between the two areas. Any difference highlighted in the univariate analysis (*p* < 0.05) was considered and systematically included in the multivariate analysis. Categorical variables were compared in the two areas using Fisher exact test, whereas quantitative variables were compared with Chi2 tests. Logistic regressions were adjusted for main potential confounders: age, sex, education level, smoking, and body mass index. Separate analyses were performed in children and adults.

## 3. Results

A total of 44 homes in the area mainly devoted to pastoralism (Filingué) and 34 in the agricultural area (Gaya) participated in the study, involving 700 subjects: 471 adults and 229 children (Table 1). The number of people per household was slightly higher in the agricultural area (15.1 ± 8.2 versus 13.9 ± 6.7), but not significantly. One-third of the households included children aged 7 to 14.

Sociodemographic characteristics of the participants were very similar in both areas. In the pastoral area, the adult population included 131 women and 125 men (mean age 34.6 years ± 16.8) and 76 girls and 49 boys (mean age 10.2 years ± 2.4). In the agricultural area, the adult population consisted of 117 women and 98 men (mean age 32.4 years ± 14.6) and 54 girls and 50 boys (mean age 10.1 years ± 2.3). Most of the individuals (91.2%) worked in agriculture (pastoralism or crop-growing). Two-thirds of the adults (resp., 62.1% in the pastoral area and 65.6% in the agricultural area) were unable to read or write. The proportion of schooled children was higher in the pastoral area than in the agricultural area (74.4% versus 56.7%; *p* = 0.005). The proportion of smokers and ex-smokers was higher among adults in pastoral areas than in agricultural areas (9.8% versus 4.2%, *p* = 0.02) (Table 1), resulting in about 19% of children exposed to environmental tobacco smoke (22.4% in the pastoral area and 14.4% in the agricultural area).

In both areas, none of the households reported the use of pesticides on crops during the period of the study. However, a large proportion of the households (87.2%) reported pesticide use at home during this period (generally only one product), in a similar proportion in the two zones. These pesticides were mainly insecticides to fight against flies and mosquitoes. Most of the households (70.9%) reported the use of the RAMBO insect powder containing permethrin that is generally spread on the floor. Other insecticides used in the households were liquids (called* pyia-pyia*) in 25.5% of households, and 3.6% were mosquito coil sprays containing permethrin and piperonyl butoxide. It was not possible to assess pesticides sprayed on crops in the agricultural area.

Of note, many villagers in the agricultural zone (Gaya) usually lit a bushfire near their homes at sunset for mosquito protection. 100% of households in both zones used wood-based biomass energy outside their homes for cooking. The proportion of adults who reported respiratory symptoms in the previous 12 months was significantly higher in the agricultural area than in the pastoral area: 43.7% versus 12.9% for wheezing (*p* < 0.0001), 65.6% versus 24.3 for dyspnea (*p* < 0.0001), and 31.6% versus 22.3% for sudden shortness of breath (*p* = 0.02) (Table 2). The frequency of asthma diagnosed by a health professional was also higher in the agricultural area (2.8% versus 1.6% in the pastoral area; *p* = 0.27), but not significantly. On the other hand, ocular and nasal irritations were reported less frequently in the agricultural area than in the pastoral area in adults (46.9% versus 62.9%; *p* = 0.0005). In children, the frequency of respiratory symptoms (wheezing, sudden shortness of breath, diagnosis of asthma, and cough) was higher in the agricultural area, but the difference was significant only for cough (32.0% versus 12.0%; *p* = 0.0002). The frequency of malaria was significantly higher in the agricultural area (*p* < 0.0001 in adults and *p* < 0.05 in children).

The majority of households were grouped in villages in both zones. This was the case for 100% in the pastoral area (Filingué), whereas 7.7% of households in the agricultural area were scattered in the fields outside the villages. There was no difference in the rate of respiratory symptoms between Gaya people living in villages and those outside among the crops (data not shown).

Results were confirmed in the multivariate analysis taking into account age, gender, tobacco smoke for adults or environmental tobacco smoke for adults and/or children, and insecticide use in the home. Adults in the agricultural area had a fourfold greater risk of wheezing than those in the pastoral area (OR = 4.64, 95% CI 2.86 to 7.54; *p* < 0.0001), a higher risk of dyspnea (OR = 2.41, 95% CI 1.63 to 3.56; *p* = 0.0001), and a higher risk of sudden shortness of breath (OR = 1.67, 95% CI 1.08 to 2.58; *p* = 0.02) and cough without fever (OR = 1.65, 95% CI 1.00 to 2.71; *p* = 0.05) (Table 3). Children in the agricultural area had a significantly increased risk of cough without fever (OR = 3.34, 95% CI 1.67 to 6.66; *p* = 0.0006). Owing to the low number of asthma sufferers, asthma was not studied in the multivariate analysis (Table 4).

## 4. Discussion

This study was designed to study respiratory health in Niger by comparing the respiratory symptoms of two populations living in pastoral and agricultural areas and to study their relationship with some environmental factors.

The risk of developing respiratory diseases and symptoms in the crop-growing area was higher among both adults (wheezing, dyspnea, sudden shortness of breath, and cough without fever) and children (cough without fever). The results remained unchanged when taking into account the main confounders, including tobacco smoke in adults and environmental tobacco smoke exposure in children.

The data from a recent review concerning asthma and COPD in Sub-Saharan Africa can be compared with ours [18]. Regarding asthma, a study in Nigeria in a representative sample of 810 adults showed a frequency of asthma diagnosed by a physician similar to that found in our survey (2.0% versus 2.1% in our study) [19]. In South Africa, a 3.7% prevalence of asthma diagnosis in men and 3.8% in women were observed [20]. Another study in Nigeria carried out among students aged 15–35 showed a 9% prevalence of wheezing and 9.4% of nocturnal cough [21], which is slightly lower than that in our study.

While the relationship between pesticide exposure and respiratory symptoms including asthma is consistently found in studies in the Northern and Mediterranean countries in both adults [22, 23] and children [24], it has not been described to date in Africa. In fact, this is the first respiratory epidemiological study in rural areas in Niger, a country whose specific climatic characteristics make comparisons difficult with the results of studies in other African countries that have a more humid climate. The only work that can be compared to ours is an Ethiopian study involving two populations: farmers applying pesticides and workers not involved in pesticide-related activity [25]. The authors observed a higher frequency of cough and wheezing among farmers in contact with pesticides. It should be noted that the Ethiopian study was conducted in an area with more rainfall than the agricultural zone of Niger.

The difference we observed in the frequency of respiratory symptoms between the two areas calls for an explanation. The use of insecticides at home does not appear to explain the increase in respiratory symptoms. Indeed, when adjusting on home insecticide use, the frequency in respiratory symptoms remained higher in the crop-growing area. Alternative explanations could be exposure to smoke from bush fires, allergenic factors related to the crops, the impact of climatic conditions, and the use of pesticides on crops that were not reported by the participants. On the contrary, the higher rate of ocular and nasal symptoms in the desert area could be due to exposure to sand.

The main strength of our study is the high comparability of the two populations according to their sociodemographic characteristics. Indeed, they differed essentially in their residential environmental and agricultural characteristics. In addition, our survey collected respiratory data at the individual level through face-to-face interviews, thereby avoiding the bias encountered in ecological studies in which some major factors like tobacco smoke cannot be taken into account. Despite the intensive training of the investigators and their integration in the community, we cannot rule out that cultural or linguistic specificities may have interfered with the understanding of the questions. However, our data are globally consistent, such as the higher prevalence of malaria in the agricultural areas owing to differences in climate (66.7% versus 48.6%; *p* < 0.0001).

Nevertheless, we encountered some difficulties in characterizing the environmental risk factors for respiratory health, the main ones in Niger being pesticides, biomass-burning, and pollen. Owing to the study conditions, we were unable to evaluate pollen exposure. Regarding biomass use which was similar in the two areas, we noticed that bush fires could be used to repel mosquitoes, specifically in the agricultural area. Surprisingly, no participant reported using pesticides on crops, whereas insecticides were widely used inside homes in both areas. However, we cannot rule out that home chemicals could also have been used on crops in the agricultural area. Indeed, they are very easy to find on the street markets, cheap, and considered to be highly active by the local populations [26].

In conclusion, this study demonstrates the necessity and the possibility of developing health surveillance programs in Niger, including information and prevention campaigns on risk factors like pesticides and the implementation of epidemiological studies. According to WHO, health research must first contribute to the well-being of populations [27]. Our study can be considered as being part of a global strategy to prevent the potentially deleterious health impact of environmental factors. Moreover, the study participants constantly expressed the need for information on health risks, which reflects their increased awareness of these issues.

## Figures and Tables

**Table 1:** Demographic characteristics of households and individuals by areas, 2013.

	Total	Pastoral area	Agricultural area	*p* ^*∗*^
*Households*	78 (100.0)	44 (56.4)	34 (43.6)	
Household size (*n* = 78)				0.07
Mean number of residents (±SD)	13.3 (±7.5)	11.9 (±6.7)	15.1 (±8.2)	
Median [min–max]	13 [2–29]	11 [2–27]	16.5 [3–29]	
% with children	32.7	32.8	32.6	
Residence on a farm (*n* = 78)				0.005
No	72 (92.3)	44 (100.0)	28 (82.4)	
Yes	6 (7.7)	0	6 (17.6)	
Type of insecticide used in the household (*n* = 55)				0.02
Rambo (powder)	39 (70.9)	28 (77.9)	11 (57.9)	
Piya-piya (liquid)	14 (25.5)	8 (22.2)	6 (31.6)	
Spray/spiral	2 (3.6)	0	2 (10.5)	
*Individuals*				
Adults	471 (100.0)	256 (54.4)	215 (45.6)	
Age (*n* = 471)				0.36
Mean (±SD)	33.5 (±15.9)	34.6 (±16.8)	32.4 (±14.6)	
Median [min–max]	30 [15–90]	30 [15–90]	30 [15–80]	
Gender (*n* = 471)				0.48
Woman	248 (52.7)	131 (51.2)	117 (54.4)	
Man	223 (47.3)	125 (48.8)	98 (45.6)	
Literate (can read and write) (*n* = 471)				0.43
No	300 (63.7)	159 (62.1)	141 (65.6)	
Yes	171 (36.3)	97 (37.9)	74 (34.4)	
Employment (*n* = 206)				0.07
At home (housewives, disabled,…)	20 (8.8)	7 (5.7)	13 (12.5)	
Farmer	206 (91.2)	115 (94.3)	91 (87.5)	
Smoke status (*n* = 471)				0.02
Never	437 (92.8)	231 (90.2)	206 (95.8)	
Former/current	34 (7.2)	25 (9.8)	9 (4.2)	
Children	229 (100.0)	125 (54.6)	104 (45.4)	
Age (*n* = 229)				0.82
Mean (±SD)	10.2 (±2.3)	10.2 (±2.4)	10.1 (±2.3)	
Gender (*n* = 229)				0.18
Girl	130 (56.8)	76 (60.8)	54 (51.9)	
Boy	99 (43.2)	49 (39.2)	50 (48.1)	
Schooled (*n* = 229)				0.005
No	77 (33.6)	32 (25.6)	45 (43.3)	
Yes	152 (66.4)	93 (74.4)	59 (56.7)	
Household smoking (*n* = 229)				0.12
No	186 (81.2)	97 (77.6)	89 (85.6)	
Yes	43 (18.8)	28 (22.4)	15 (14.4)	

Quantitative variable: mean, SD, median, minimum, and maximum.

Qualitative variable: number and percentage.

^*∗*^Wilcoxon test or Student's *t*-test for quantitative variables and Chi2 test or Fisher for qualitative variables.

**Table 2:** Frequency of health events reported by subjects, 2013.

		Adults (*N* = 471)				Children (*N* = 229)			
		Total (%)	Pastoral (*n* = 256)	Agricultural (*n* = 215)	*p*	Total (%)	Pastoral (*n* = 125)	Agricultural (*n* = 104)	*p*
Asthmatics symptoms									
Asthma confirmed by health worker	Yes	2.1	4 (1.6)	6 (2.8)	0.27	3.9	3 (2.4)	6 (5.8)	0.16
Wheezing	Yes	26.9	33 (12.9)	93 (43.2)	<0.0001	27.6	32 (25.6)	31 (29.8)	0.44
Dyspnea	Yes	54.4	114 (24.3)	141 (65.6)	<0.0001				
Sudden shortness of breath	Yes	26.5	57 (22.3)	67 (31.2)	0.02	23.6	25 (20.0)	29 (27.9)	0.16
Other respiratory symptoms									
Cough	Yes	33.7	84 (32.8)	74 (34.4)	0.65	35.8	42 (33.6)	40 (38.5)	0.41
Awakened by a coughing fit	Yes	42.2	110 (43.0)	87 (40.5)	0.70	34.9	43 (34.4)	37 (35.6)	0.85
Cough without fever	Yes	17.3	38 (14.9)	43 (20.0)	0.13	21.1	15 (12.0)	33 (31.7)	0.0002
Eye and nasal symptoms									
Eye irritation	Yes	55.7	162 (62.9)	100 (46.5)	0.0005	52.0	75 (60.0)	43 (41.3)	0.007
Nasal irritation without cold	Yes	33.1	90 (35.2)	65 (30.2)	0.30	40.8	52 (41.6)	41 (39.4)	0.78
Malaria	Yes	56.8	124 (48.6)	142 (66.0)	<0.0001	74.4	86 (69.4)	83 (79.8)	0.05

**Table 3:** Relationship of respiratory symptoms in adults (multivariate analysis), 2013.

Symptoms	Pastoral (*n* = 256)	Agricultural (*n* = 215)	Adjustment variable
	*n* (%), OR	*n* (%), OR (95% CI)	
Wheeze	33 (12,9), 1.00	93 (43.2), 4.64 (2.86; 7.54)^*∗∗∗*^	Age, gender, marital status, insecticide use in the home
Dyspnea	114 (44.5), 1.00	141 (65.6), 2.41 (1.63; 3.56)^*∗∗∗*^	Age, gender, marital status
Sudden shortness of breath	57 (22,3), 1.00	67 (31.2), 1.67 (1.08; 2.58)^*∗*^	Age, gender, marital status
Cough	84 (32,8), 1.00	74 (34.4), 1.14 (0.75; 1.72)	Gender, literate, insecticide use in the home
Awakened by coughing	110 (43.0), 1.00	87 (40.5), 0.86 (0.59; 1.26)	Gender, marital status
Cough without fever	38 (14,9), 1.00	43 (20.0), 1.65 (1.00; 2.71)^*∗*^	Age, insecticide use in the home
Eye irritation	162 (62.9), 1.00	100 (46.5), 0.53 (0.35; 0.80)^*∗∗*^	Age, insecticide use in the home
Nasal irritation without cold	90 (35.1), 1.00	65 (30.2), 0.77 (0.52; 1.15)	Age

*n*: number of subjects. ^*∗*^*p* < 0.05, ^*∗∗*^*p* < 0.001, and ^*∗∗∗*^*p* < 0.0001.

**Table 4:** Relationship respiratory events in children (multivariate analysis), 2013.

Symptoms	Pastoral (*n* = 125)	Agricultural (*n* = 104)	Adjustment variable
	*n* (%), OR	*n* (%), OR (95% CI)	
Wheeze	32 (25.6), 1.00	31 (29.8), 1.38 (0.66; 2.86)	Age, gender, insecticide use in the home
Sudden shortness of breath	25 (20.0), 1.00	29 (27.9), 1.44 (0.79; 2.63)	Age, insecticide use in the home
Cough	42 (33.6), 1.00	40 (38.5), 1.48 (0.82; 2.67)	Age, insecticide use in the home
Awakened by coughing	43 (34.4), 1.00	37 (35.6), 1.13 (0.64; 1.98)	Age, insecticide use in the home
Cough without fever	15 (12.0), 1.00	33 (31.7), 3.34 (1.67; 6.66)^*∗∗∗*^	Age, presence of smoking in the house
Eye irritation	75 (60.0), 1.00	43 (41.3), 0.50 (0.29; 0.86)^*∗*^	Gender
Nasal irritation without cold	52 (41.6), 1.00	41 (39.4), 0.99 (0.57; 1.71)	Insecticide use in the home

*n*: number of subjects. ^*∗*^*p* < 0.05 and ^*∗∗∗*^*p* < 0.0001.
